# Anticonvulsant hypersensitivity syndrome after phenytoin administration in an adolescent patient: a case report and review of literature

**DOI:** 10.1186/s12948-017-0069-0

**Published:** 2017-06-15

**Authors:** Malik Ghannam, Shaden Mansour, Aya Nabulsi, Qusay Abdoh

**Affiliations:** 1 AN-Najah National University Teaching Hospital, Asira, Nablus, West bank Palestine; 2 AN-Najah National University, Nablus, West bank Palestine

**Keywords:** Phenytoin, Hypersensitivity, Adolescent patient, Rare condition, DRESS, Case report, Literature review

## Abstract

**Background:**

Hypersensitivity is a rare adverse drug reaction (ADR) associated with anti-epileptic medications. Phenytoin is one of the commonly used drugs for treatment of epilepsy that encounters a hypersensitivity reaction. This reaction can be ranged from mild cutaneous rash to anticonvulsant hypersensitivity syndrome (AHS) or drug reaction with eosinophilia and systemic symptoms (DRESS) that includes fever, rash, eosinophilia and involvement of multiple internal organs.

**Case presentation:**

A 15 year old middle eastern female patient from Gaza strip with free past medical and allergic history. She presented to An-Najah National University Hospital (NNUH) in Nablus with intermittent high grade fever, jaundice, rash and skin peeling. On examination, she had axillary and inguinal lymphadenopathy, moderate splenomegaly and diffuse maculopapular rash. The patient was on phenytoin which started 1 month prior to her presentation as a seizure prophylaxis due to previous head injury. Eventually, the patient was diagnosed with AHS/DRESS.

**Conclusions:**

AHS is a diagnosis of exclusion and it is significantly underreported that requires a high index of suspicion. We liked to share this case and shed the light in more details on AHS/DRESS. Our goal was to help making AHS more reported in the literature in adolescent patients, as well as to make physicians more alert of this condition’s seriousness when they prescribe antiepileptic medications in particular. In this report, we included the first case of AHS which was reported in an adolescent patient in Palestine. Moreover, we reviewed the available literature for a better understanding of the pathophysiology and management of AHS. We still believe that the full understanding of the pathogenesis of AHS is lacking, and also we are lacking a clinical tool or scoring system to determine the severity of AHS/DRESS.

## Background

Anticonvulsant hypersensitivity syndrome (AHS) is a rare, potentially fatal, systemic and idiosyncratic drug-induced reaction. The syndrome has been reported with aromatic antiepileptic drugs (AEDS); such as phenytoin, carbamazepine and phenobarbital [1]. AHS is a triad of fever, rash, and internal organ involvement. Fever in conjunction with pharyngitis and weakness is usually the first complaint [2].

AHS is a multi-organ condition with a mortality rate of 10–20% [3]. It was described for the first time in 1950s [4] while the antiepileptic reaction of phenytoin was first recognised by meritt and putnam early in 1930s [5]. This hypersensitivity reaction primarily associated with phenytoin, carbamazepine, lamotrigine, and phenobarbital administration [6]. In most cases, the condition appears within 2 weeks to 2 months after exposure to the anti-epileptic medication [7]. It is estimated to occur in one every 1000–10,000 exposures [8]. Since most of the cases are underreported and unrecognized, its true incidence still unknown [6]. Previous evidence suggested that there is no gender variation in the development of AHS [2]. Because many recent studies conclude that women especially fertile women are more prone to this drug reaction, hormonal phenomenon might be included in the pathogenesis of AHS [9]. The majority of the reported cases has been in african american patients [7]. It is worth mentioning that AHS has an autosomal dominant inheritance pattern; with increased susceptibility between siblings of affected patients [10]. A high rate of cross-reactivity among aromatic anti epileptic drugs (AEDS) is of concern and may be as high as 80% [10]. AHS is previously known as drug reaction with eosinophilia and systemic syndrome (DRESS) [9].

Although the exact mechanism of AHS remains not well understood, genetic factors may predispose to its occurrence. The human leukocyte antigen HLA-A*3101 was found to be associated with the carbamazepine cutaneous reaction in patients with the eurapean origin [4]. Human herpesvirus 6 (HHV6) and /or human herpesvirus 7 (HHV7), CMV and EBV are all associated with AHS [3]. Reactivation of HHV6 was particularly linked to AHS/DRESS, which may be related to reduced CD19B cells [11] and enhanced T helper cell type 2 activity that occurs in HHV6 reactivation [12]. While, some studies conclude that anti-epileptic medications may resemble viral infection by enhancing the CD4 and CD8 T Cells activity resulting in increase of IL5, which play a major role in the maturation of the eosinophils [13]. AHS is generally thought to be a type IV T cell mediated delayed hypersensitivity reaction [6]. However, it is postulated that anti-epileptic medications containing aromatic ring are metabolized to arene oxide metabolites by hepatic cytochrome P450. Defective detoxification and elimination have been identified in patients with AHS. The accumulation of these metabolites stimulates cell necrosis and immunological response by binding to cellular macromolecules which explains AHS symptoms [10].

In this article, we report an interesting case of AHS 1 month after phenytoin administration with a significant hepatic injury in an adolescent patient in Palestine.

## Case presentation

A 15 year old middle eastern female patient from Gaza strip with free past medical and allergic history who was admitted at our internal medicine department on July 20. 2016. At time of admission, she had intermittent high grade fever and sore throat for 1 week duration. Additionally, a non-itchy skin eruption was noticed for 2 weeks duration. As well as she started to have yellowish eye discoloration 5 days after the onset of fever. The patient was taking phenytoin 200 mg QD PO as a seizure prophylaxis since she had significant head trauma 4 weeks prior to her presentation.

She denied any history of red eye, digital swelling, seizures, photophobia or any weight changes. Family history was unremarkable. She was sexually inactive and started to have irregular menstruation 1 year before the time of admission. In addition to phenytoin, her only medication at the time of admission was acetaminophen 500 mg twice daily.

**Fig. 1:**
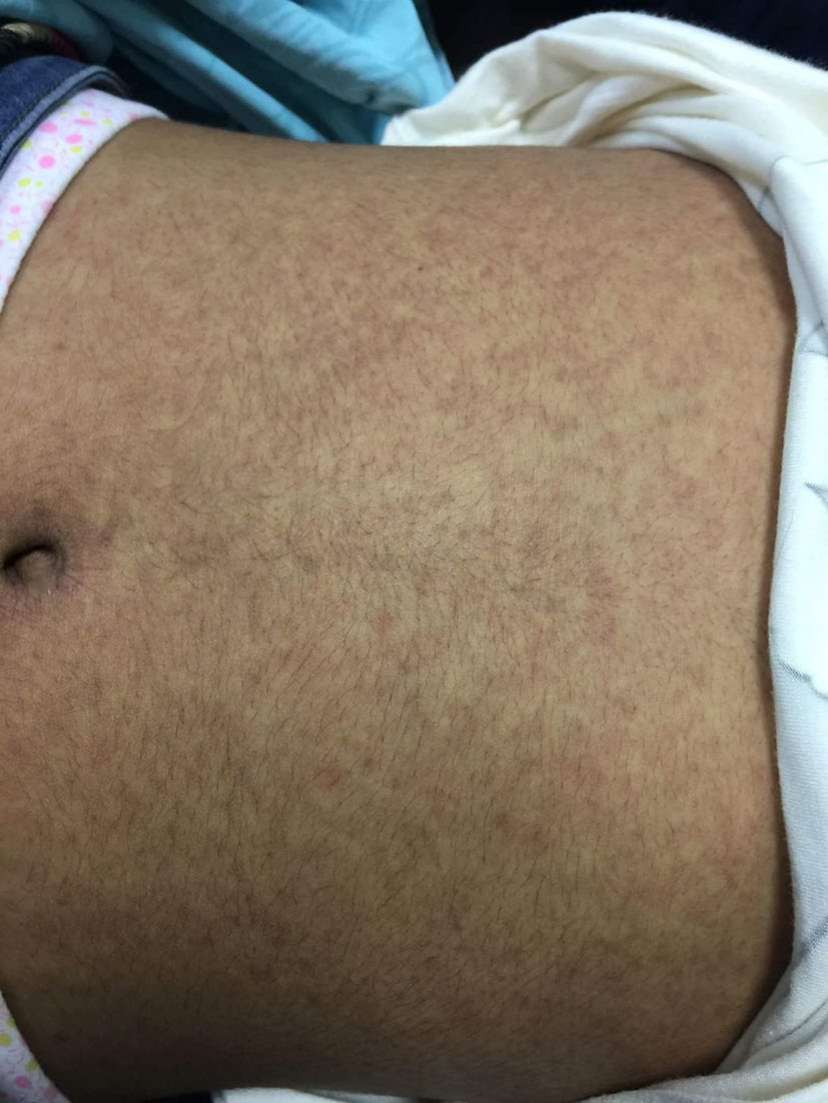
Erythematous maculopapular rash on the abdomen

**Fig. 2:**
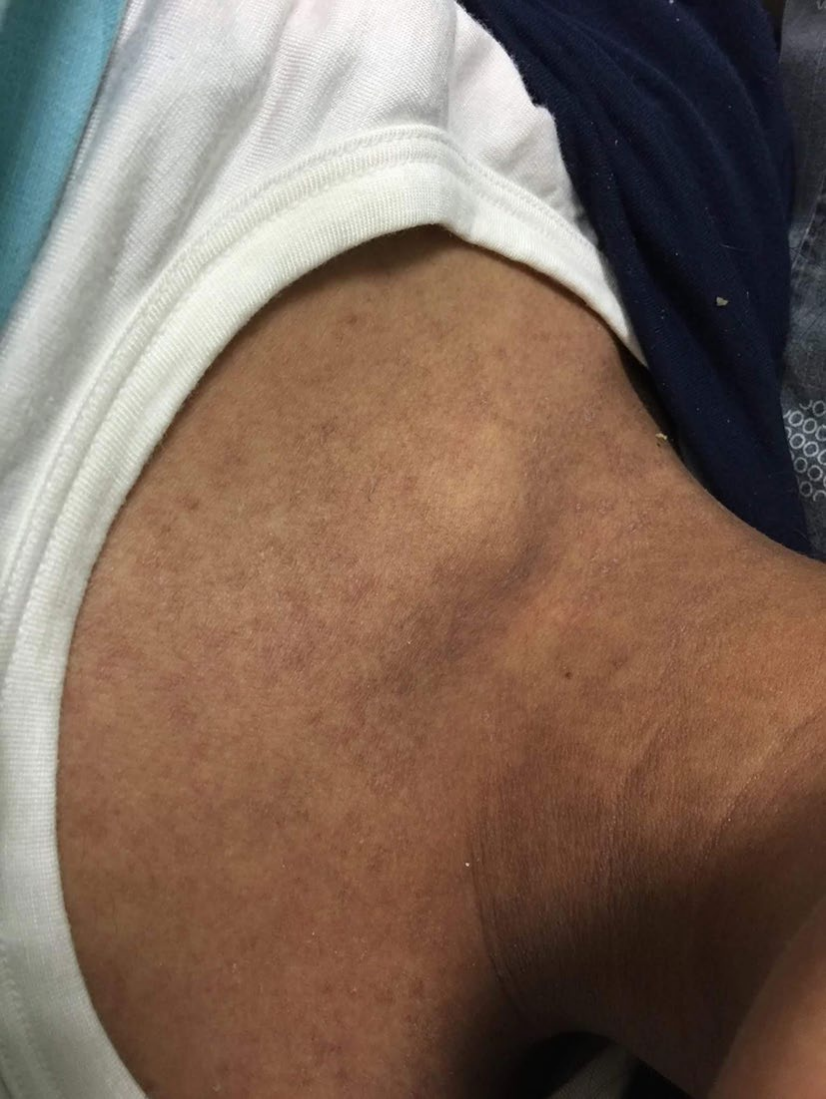
Erythematous maculopapular rash over the neck

**Fig. 3:**
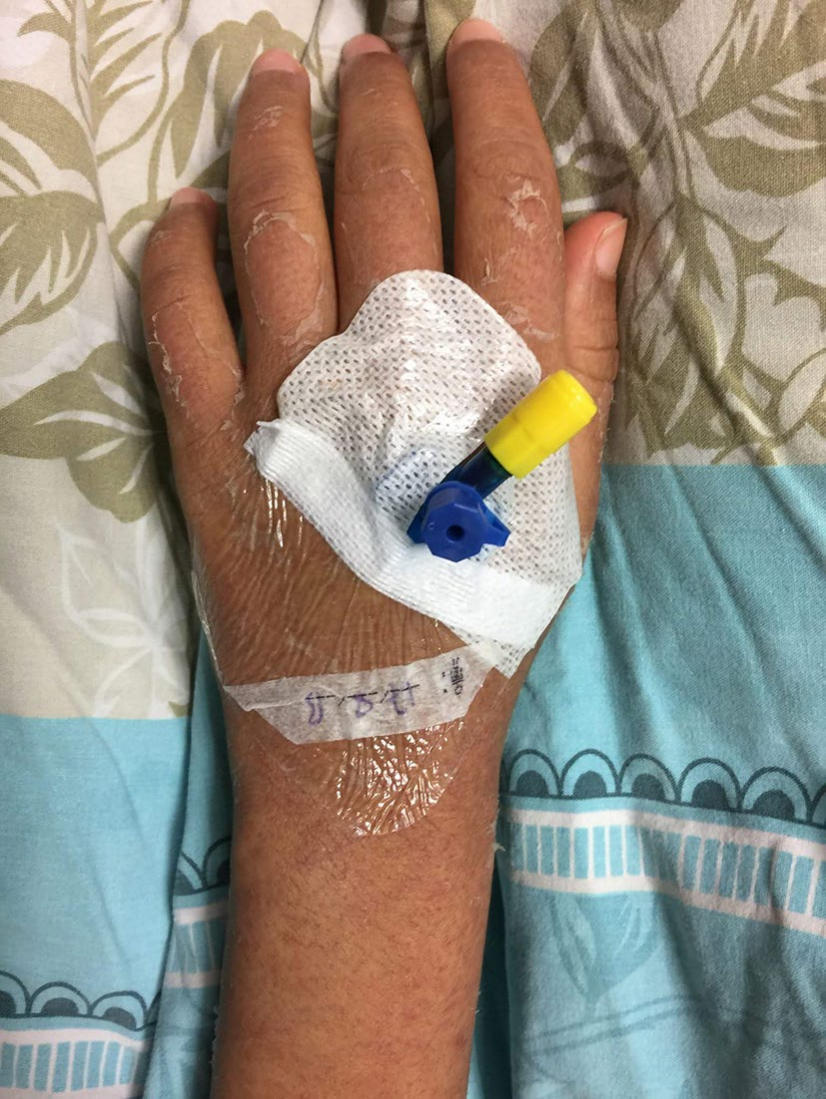
Hand maculopapular rash with desquamation

**Fig. 4:**
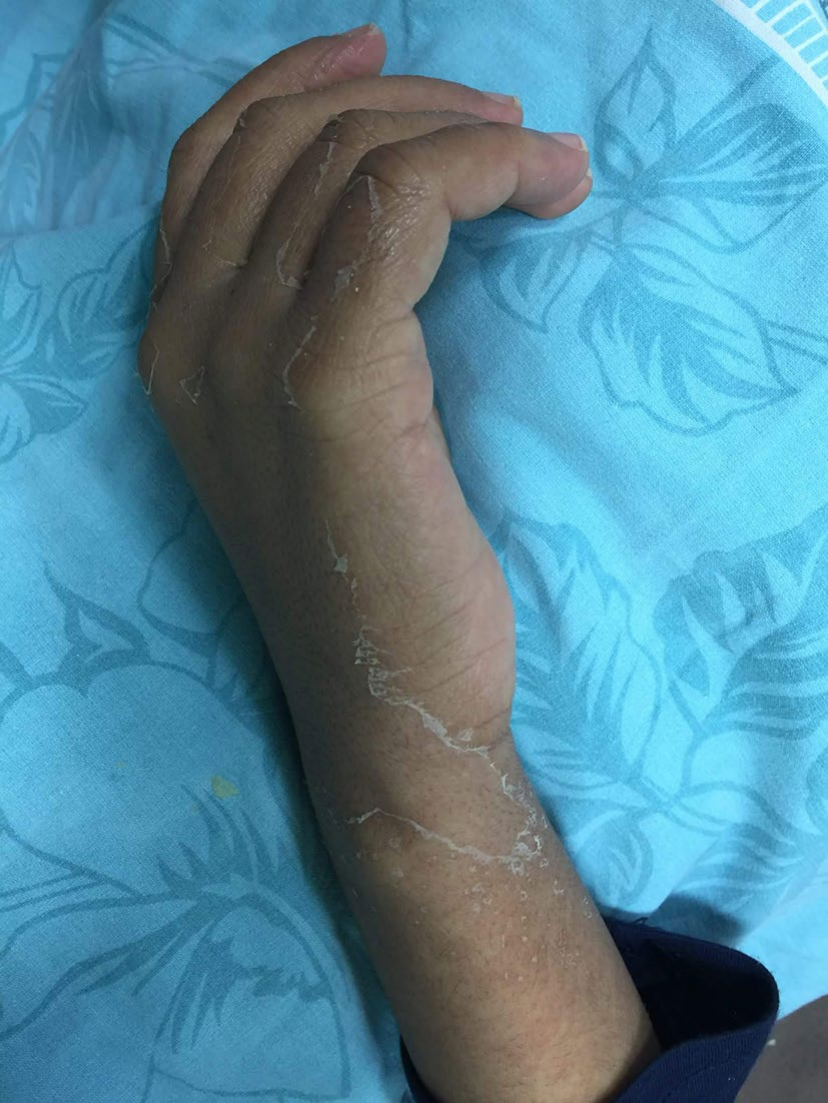
Hand skin peeling

On physical examination, she was alert, oriented and conscious. Her hemodynamics were stable. Temperature 39.9 C and her tonsils were erythematous. Generally, she looked deeply jaundiced as well as fatigued. Systemic examination was normal except maculopapular rash on her limbs, abdomen, chest and back (Figs. 1, 2) besides peeling on both palms (Figs. 3, 4). On abdominal examination, the spleen was palpable 2 cm below costal margin and her liver was palpable and tender as well. She also had left axillary and inguinal lymphadenopathy around 1 × 1 cm in diameter each.

**Fig. 5:**
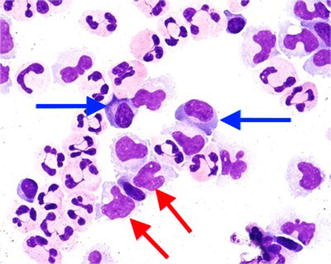
Atypical lymphocytes on peripheral blood smear

Abdominal ultrasound was done and showed moderate hepatosplenomegaly, in addition to that, there was enlarged retroperitoneal, portahepatis and mesenteric lymph nodes. She also underwent brain MRI which did not show any intracranial pathology. Liver Doppler and cardiac echocardiography were both normal. Chest x-ray and EKG were normal as well. Laboratory work up was summarized in Table 1.

**Table 1:** Laboratory findings at both times of admission and discharge

Initial findings at time of admission		
Hemoglobin 10.4 g/dl	Albumin 3.1 g/dl	Prothrombin time 11.7 s, partial thromboplastin time 29 s
Platelets 105,000/mm^3^	Alkaline phosphatase 459 U/l	Anti-smooth muscle and anti-liver kidney microsomal antibodies were negative
White blood cells 10.45 × 10^3^/μl	Gamma-glutamyl transferase 964 U/l	Hepatitis profile was all negative
Neutrophils 4.83 × 10^3^/μl	Total bilirubin 4.3 mg/dl	EBV & CMV IgMs were negative, monospot test was negative as well
Lymphocytes 3.80 × 10^3^/μl	Direct bilirubin 4 mg/dl	Respiratory viral panel was negative
Eosinophil 1.60 × 10^3^/μl	Creatinine 0.72 mg/dl	ANA, anti-dsDNA and anti-histone were all negative
Alanine aminotransferase 1198 IU/l	CRP 4.7 mg/dl	Urine analysis, urine culture, blood culture, stool culture and throat swap culture were all negative
Aspartate Aminotransferase 1338 IU/l	Erythrocytes sedimentation rate 27 mm/h	Blood film showed atypical lymphocytes (Fig. 5)
10 days later at time of discharge		
Hemoglobin 10.9 g/dl		
Total bilirubin 2.07 mg/dl		
Direct bilirubin 1.68 mg/dl		
Aspartate aminotransferase 31 IU/l		
Alanine aminotransferase 96 IU/l		
White blood cells 9.7 × 10^3^/μl		
Platelets 288,000/mm^3^		

Beside AHS, autoimmune hepatitis and infectious mononucleosis were suspected at that time. Phenytoin was stopped as well as acetaminophen due to elevated liver enzymes. The patient was given IV hydrocortisone. Over 10 days of admission, her condition significantly improved then she was sent home. The patient was seen 1 month after her discharge, at that time, the fever was gone, the rash was resolved and her liver enzymes were normalized.

## Discussion

AHS is mainly characterized by high intermittent fever, skin rash, eosinophilia and internal organ abnormalities [14]. Fever is the most common manifestation occurring in approximately 90–100% of exposures. Commonly, fever and malaise are the first manifestations [4]. Skin rash is seen in 90% of the AHS cases, which is mainly a maculopapular eruption with subsequent desquamation during resolution [1]. Moreover, the rash can be ranged from morbilliform eruption to more severe forms like Steven Johnson syndrome or toxic epidermal necrosis [15]. The incidence of these two syndromes is about 9% in AHS patients [16]. The cutaneous reaction to anticonvulsants medications is typically known as pseudolymphoma due to the similarities in histology and clinical presentation with malignant lymphoma [17]. Skin biopsy usually reveals T lymphocyte infiltration and tissue eosinophilia [18, 19]. Noticeably, carbamazepine is associated with a higher incidence of skin reaction compared to phenytoin and lamotrigine. Whereas valproic acid and topiramate are rarely associated with skin manifestation [9]. Table 2 summarizes the adverse reactions associated with some anticonvulsant medications.

**Table 2:** Adverse reactions associated with anticonvulsant medications

Drug	Drug reaction
Phenytoin	Most common cause of AHS, other reactions include: erythroderma, facial pustules, hyperpigmentation, hypertrichosis and lupus-like symptoms
Carbamazepine	Oral ulcers, urticaria, Stevens-Johnson and photosensitivity
Fosphenytoin	Bullous rash, erythroderma, pruritus, gingival hyperplasia, lupus-like symptoms and erythema multiforme
Lamotrigine	Hives, swollen glands, painful sores in or around eyes or mouth, difficulty breathing, swelling of face, lips, tongue or throat

Systemic involvement is the major cause of mortality and morbidity in AHS. Liver is the most frequently affected internal organ occurring in 50–60% of cases [14]. Hepatic involvement could be just an elevation of the liver enzymes level up to fulminant hepatic failure [20]. Hematological manifestation as leukocytosis with atypical lymphocytes and increased eosinophil are seen in 50% of patients [1]. Other possible abnormalities like carditis, pneumonitis, conjunctivitis, thrombocytopenia, splenomegaly, myalgia and hemolytic anemia can be seen as well [14]. Delayed hypothyroidism for several months after the acute phase has been described in AHS [18]. Rare manifestation like hypersensitivity colitis can be found in this condition, thereby AHS patients may present with diarrhea [21, 22]. Additionally, periorbital or orofacial edema might also be expected [23]. The main organs which are involved in AHS are epidermis in the skin, clara cell in the lungs, liver and tubular cells in the kidney. All of these cells possess cytochrome P450, which is responsible for metabolism of anti-epileptic medications.

Although reduced immunoglobulins (IgA, IgM, IgG) is noticed at the onset of the hypersensitivity response, several weeks after the suspension of the anti-epileptic medication the immunoglobulins level start to increase [11, 18, 24]. While, several tests used to confirm the diagnosis of AHS, like patch testing and lymphocytes transformation test which is used to assess the drug reactive T cells [18], AHS remains a diagnosis of exclusion which is largely achieved by the following: clinical features, absence of a septic focus and the probable response to steroid treatment [7]. Table 3 summarizes the clinical features of AHS.

**Table 3:** The clinical features of anticonvulsants hypersensitivity syndrome

Finding	Incidence %
Fever	90–100
Skin rash	90
Liver involvement	50–60
Hematological manifestation	50

The most critical component of management of the AHS is the suspension of all potentially causative anti-epileptic medications [8]. All cases with hypersensitivity must be hospitalized even if the clinical presentation is mild [18]. Mild cases treated with supportive care [18]. The conventional treatment for AHS is systemic glucocorticoides 1–2 mg/kg/day [25] or 40–60 mg/day. The drug of choice is Prednisolone [26], that should be carefully tapered over 6–8 weeks to minimize the chances of relapse [18]. Glucocorticoides, anti-histamines (H1-receptor blockers), epinephrine and airway management all can be used depending on the severity and the extent of the condition [15]. If systemic steroid is not sufficient to alleviate the symptoms, other treatment options are available including plasmapheresis, IVIG [18] and N-acetylcysteine [10]. IVIG has the capability to block the fc receptor of the immunoglobulins by forming different immune complexes and decreasing the inflammatory response. While N-acetylcysteine is solely used in case of hepatic and dermatological compromise, it can buffer the toxic metabolites as well as it has an antiapoptotic effect. In addition to that, N-acetylcysteine has the ability to limit the production and expression of various cytokines and molecules like keratinocyte intercellular adhesion molecule 1 (ICAM-1), that are implicated in the inflammatory cascade of AHS.

The use of antibiotics and NSAIDs carries no benefit in the treatment of AHS, particularly in the acute phase. Moreover, antibiotics and NSAIDs may worsen or even mask the clinical picture of AHS due to the cross reactivity [18]. In patient requiring ongoing anti-epileptic treatment, sodium valproate considered to be a safe alternative because of its different chemical structure [6]. Careful monitoring of thyroid function test for several months would be valuable in AHS due to the expected late onset hypothyroidism during the course of this reaction.

Although it was postulated that the prognosis of AHS determined by various factors such as; genetic predisposition, patient age, reactivation of some viruses like HHV6, underlying disease process and the type of management [27], we are lacking an accurate clinical tool or scoring system to determine AHS/DRESS prognosis [28].

AHS is a synonymous for drug reaction with eosinophilia and systemic symptoms (DRESS). It is also known as drug induced hypersensitivity syndrome (DIHS), hypersensitivity syndrome (HSS) and drug induced delayed multiorgan hypersensitivity syndrome (DIDMOHS). DRESS is a wider term than AHS that caused by failure of detoxification of several medications [28], including, phenytoin, carbamazepine, phenobarbital, zonisamide, mexiletine, lamotrigine, abacavir, nevirapine, dapsone, salazosulfapyridine, allopurinol and minocycline [29]. Although one of the diagnostic criteria of Japanese consensus group to diagnose DRESS is to have reactivation of human herpesvirus 6 (HHV6) [28], the initial HHV6 serological testing for our patient was negative. According to Shiohara et al. DRESS or DIHS pathogenesis is a profound interactions between herpesviruses reactivation (HHV6, HHV7, CMV, EBV), immune response to these viruses and specific immune response to the culprit medication itself [18]. The clinical diagnostic criteria of DRESS based on RegiSCAR group was summarized in Table 4 which fits our patient’s diagnosis with AHS [28, 29].

**Table 4:** The clinical criteria for DRESS by RegiSCAR group

Fever >38 °C
Enlarged lymph nodes at least of 2 sites
Involvement of at least one internal organ
Blood count abnormalities, at least one present. Lymphocytes above or below normal, low platelets and/or eosinophilia
Hospitalization
Reaction suspected to be drug related
Acute rash

At least three of the first four criteria are required to make the diagnosis

There are very limited differentials for thousands elevation of liver enzymes as we encountered in our patient. We thought of viral and autoimmune hepatitis at the time of admission. Viral hepatitis was ruled out by negative hepatitis profile. Autoimmune hepatitis (AIH) was kept in mind. AIH was first described in 1950s by Waldenström [30]. The clinical presentation is variable ranging from no obvious signs or symptoms to acute or even fulminant liver disease [31]. AIH affects females more frequently than males with a ratio 3.6:1. Although there is no race predilection in AIH [32], but the age distribution is bimodal, one peak during teenage years and another in fourth and sixth decade of life [31]. AIH is classified into two types. Type one is characterized by the presence of ANAs and/or anti smooth muscle antibodies (ASMAs). Whereas the less common type two is defined by positivity for anti-liver-kidney microsomal type 1 antibody (anti-LKM1) or anti liver cytosol type 1 antibody (anti-LC 1) [30]. Our patient was tested for the aforementioned antibodies and they were all negative.

A systemic disease like infectious mononucleosis may present with similar manifestations and clinical course as our patient. Infectious mononucleosis is a clinical syndrome caused by EBV and commonly presents in adolescents and children. Based on Hoagland’s criteria [33] for EBV infectious mononucleosis, to make the diagnosis, we require at least 50 percent lymphocytes and at least 10 percent atypical lymphocytes in the presence of fever, adenopathy and pharyngitis. The diagnosis confirmed by a positive serologic investigation. Talking about the hematological findings, although the CBC and blood smear of our patient showed lymphocytosis with atypical lymphocytes which are also seen in infectious mononucleosis, it is unlikely to have eosinophilia in infectious mononucleosis as our patient had. However, our patient does not fit the aforementioned criteria for EBV infectious mononucleosis [34]. Despite of the negative serological testing in our patient, reactivation of EBV, CMV, HHV 6 and HHV 7 were also detected in DRESS by running longitudinal studies using PCR to test viral DNA [28].

Adult onset still’s disease (AOSD) can mimic the clinical findings of AHS. AOSD is a rare systemic inflammatory disease. It has been described all over the world and has a bimodal age distribution; the first peak occurs at 15–25 years of age and the second one usually occurs within 36–46 years. It is characterized by the classical triad of persistent high spiking fever, arthralgia and salmon colored skin rash. AOSD is typically a diagnosis of exclusion and should be considered after excluding infectious, malignant and other connective tissue diseases. Our patient does not fit the typical presentation of AOSD [35].

It is uncommon for Kawasaki disease to present in our patient age. However, it would be more significant to think of Kawasaki if the patient presented earlier. It is a clinical diagnosis which occurs mainly below age of 5 years [36]. Clinical Criteria for Kawasaki disease diagnosis are summarized in Table 5. Transthoracic Echocardiography was done in our patient to rule out coronary aneurysm, which did not show any significant finding.

**Table 5:** Clinical features for diagnosing Kawasaki disease

Classical Kawasaki	Classical Kawasaki with alternative diagnostic criteria	Atypical Kawasaki
Constant fever of at least 5 days and at least 4 of the principle features	Constant fever of at least 5 days, two or three of principle features, coronary abnormalities on transthoracic echocardiography	Constant fever of at least 5 days, two or three of the principle features
Principle features of Kawasaki disease diagnosis		
Changes in the oral cavity and lips like strawberry tongue, erythematous lips (96.5%)		
Polymorphous rash (96%)		
Bilateral non-purulent conjunctivitis (89%)		
Changes in the extremities like erythema, desquamation in hands and feet in week 2 and 3 (75.6%)		
Cervical lymphadenopathy more than 1.5 cm in diameter, mainly unilateral (62.7%)		

## Conclusions

AHS is a diagnosis of exclusion and it is significantly underreported which requires a high index of suspicion. We liked to share this case and shed the light in more details on AHS/DRESS. Our goal was to help making AHS more reported in the literature in adolescent patients, as well as to make physicians more alert of this condition’s seriousness when they prescribe antiepileptic medications in particular. In this report, we included the first case of AHS which was reported in an adolescent patient in Palestine. Moreover, we reviewed the available literature for a better understanding of the pathophysiology and management of AHS. We still believe that the full understanding of the pathogenesis of AHS is lacking, and also we are lacking a clinical tool or scoring system to determine the severity of AHS/DRESS.

## Abbreviations

AHS: anticonvulsant hypersensitivity syndrome
ADR: adverse drug reaction
AEDS: antiepileptic drugs
LP: lumber puncture
EBV: Epstein Barr virus
CMV: cytomegalovirus
HSV: Herpes simplex virus
DRESS: drug reaction with eosinophilia and systemic syndrome
HLA: human leukocyte antigen
IL: interleukin
IVIG: intravenous immunoglobulin
ICAM-1: keratinocyte intercellular adhesion molecule 1
NSAIDs: non steroidal anti-inflammatory drugs
ANAs: antinuclear antibodies
ASMAs: anti smooth muscle antibodies
Anti-LKM1: anti liver-kidney microsomal type 1 antibody
Anti-LC 1: anti liver cytosol type 1 antibody
AOSD: adult onset still’s disease
NNUH: An-Najah National University Hospital
AIH: autoimmune hepatitis
DRESS: drug reaction with eosinophilia and systemic symptoms
DIHS: drug induced hypersensitivity syndrome
HSS: hypersensitivity syndrome
DIDMOHS: drug induced delayed multiorgan hypersensitivity syndrome
HHV6: human herpesvirus 6
HHV7: human herpesvirus 7
